# Supplementary material for: Metagenomic analysis of fungal assemblages at a regional scale in high-altitude temperate forest soils: alternative methods to determine diversity, composition and environmental drivers
Source: PeerJ. 2025 Jan 2;13:e18323. doi: 10.7717/peerj.18323 (PMC11700497; doi:10.7717/peerj.18323)
Supplement: Supplemental Information 1 [file peerj-13-18323-s001.docx]

**Supplementary material**

**Metagenomic analysis of fungal assemblages at a regional scale in high-altitude temperate forest soils:** **alternative methods to determine diversity, composition and environmental drivers**

Stephanie E Hereira- Pacheco, Itzel Arias-Del Razo, Alejandra Miranda-Carrazco, Arturo Estrada Torres, Yendi E Navarro-Noya

**Table S1.** Coordinates at the start, middle, and endpoints of the 36 transects from the 12 sampling sites within 6 polygons in the study area.

| **Pol** | **Site** | **Point** | **Transect 1** | **Transect 2** | **Transect 3** |
| --- | --- | --- | --- | --- | --- |
| 1 | 1.Tetlanohcan 1 | Start | N 19°14.635' W 98°06.599' | N 19°14.741' W 98°06.551' | N 19°14.848' W 98°06.732' |
| 1 | 1.Tetlanohcan 1 | Middle | N 19°14.667' W 98°06.604' | N 19°14.749' W 98°06.559' | N 19°14.849' W 98°06.744' |
| 1 | 1.Tetlanohcan 1 | End | N 19°14.6' W 98°06.604' | N 19°14.753' W 98°06.573' | N 19°14.855' W 98°06.756' |
| 1 | 2.Tetlanohcan 2 | Start | N 19°14.736' W 98°05.606' | N 19°14.732' W 98°05.660' | N 19°14.902' W 98°05.603' |
| 1 | 2.Tetlanohcan 2 | Middle | N 19°14.738' W 98°05.603' | N 19°14.741' W 98°05.651' | N 19°14.899' W 98°05.582' |
| 1 | 2.Tetlanohcan 2 | End | N 19°14.755' W 98°05.602' | N 19°14.751' W 98°05.651' | N 19°14.900' W 98°05.573' |
| 2 | 3. La Ascención Huitzcolotepec | Start | N 19°22.032' W 98°13.945' | N 19°22.000' W 98°13.975' | N 19°21.968' W 98°14.018' |
| 2 | 3. La Ascención Huitzcolotepec | Middle | N 19°22.033' W 98°13.932' | N 19°22.014' W 98°13.974' | N 19°21.978' W 98°14.020' |
| 2 | 3. La Ascención Huitzcolotepec | End | N 19°22.030' W 98°13.943' | N 19°22.026' W 98°13.976' | N 19°21.989' W 98°14.019' |
| 2 | 4. Tepeticpac | Start | N 19°21.779' W 98°14.756' | N 19°21.816' W 98°14.807' | N 19°21.741' W 98°14.799' |
| 2 | 4. Tepeticpac | Middle | N 19°21.781' W 98°14.759' | N 19°21.801' W 98°14.807' | N 19°21.750' W 98°14.804' |
| 2 | 4. Tepeticpac | End | N 19°21.803' W 98°14.762' | N 19°21.793' W 98°14.807' | N 19°21.765' W 98°14.810' |
| 3 | 5. El Carmen Las Carrozas | Start | N 19°27.257' W 98°21.308' | N 19°27.303' W 98°21.281' | N 19°27.136' W 98°21.347' |
| 3 | 5. El Carmen Las Carrozas | Middle | N 19°27.274' W 98°21.305' | N 19°27.289' W 98°21.283' | N 19°27.146' W 98°21.332' |
| 3 | 5. El Carmen Las Carrozas | End | N 19°27.280' W 98°21.294' | N 19°27.281' W 98°21.288' | N 19°27.153' W 98°21.322' |
| 3 | 6. La Caridad | Start | N 19° 25.261' W 98°21.640' | N 19°25.303' W 98°21.622' | N 19°25.340' W 98°21.655' |
| 3 | 6. La Caridad | Middle | N 19°25.270' W 98°21.648' | N 19°25.302' W 98°21.636' | N 19°25.352' W 98°21.656' |
| 3 | 6. La Caridad | End | N 19°25.287' W 98°21.656' | N 19°25.307' W 98°21.649' | N 19°25.366' W 98°21.658' |
| 4 | 7.Tandi-Chala | Start | N 19°28.267' W 98°28.621' | N 19°28.253' W 98°28.542' | N 19°28.168' W 98°28.389' |
| 4 | 7.Tandi-Chala | Middle | N 19°28.271' W 98°28.599' | N 19°28.243' W 98°28.529' | N 19°28.172' W 98°28.402' |
| 4 | 7.Tandi-Chala | End | N 19°28.276' W 98°28.596' | N 19°28.247' W 98°28.520' | N 19°28.179' W 98°28.412' |
| 4 | 8. San Francisco Mitepec | Start | N 19°27.455' W 98°27.608' | N 19°27.378' W 98°27.625' | N 19°27.366' W 98°27.593' |
| 4 | 8. San Francisco Mitepec | Middle | N 19°27.457' W 98°27.622' | N 19°27.382' W 98°27.635' | N 19°27.361' W 98°27.610' |
| 4 | 8. San Francisco Mitepec | End | N 19°27.462' W 98°27.634' | N 19°27.386' W 98°27.649' | N 19°27.357' W 98°27.620' |
| 5 | 9. Piedra Canteada de San Felipe, Hidalgo | Start | N 19°27.270' W 98°36.034' | N 19°27.400' W 98°35.939' | N 19°27.399' W 98°35.815' |
| 5 | 9. Piedra Canteada de San Felipe, Hidalgo | Middle | N 19°27.285' W 98°36.036' | N 19°27.395' W 98°35.945' | N 19°27.410' W 98°35.821' |
| 5 | 9. Piedra Canteada de San Felipe, Hidalgo | End | N 19°27.306' W 98°36.033' | N 19°27.396' W 98°35.961' | N 19°27.424' W 98°35.823' |
| 5 | 10. Paraje El Madroño | Start | N 19°28.630' W 98°35.254' | N 19°28.611' W 98°35.340' | N 19°28.611' W 98°35.395' |
| 5 | 10. Paraje El Madroño | Middle | N 19°28.626' W 98°35.268' | N 19°28.603' W 98°35.347' | N 19°28.598' W 98°35.387' |
| 5 | 10. Paraje El Madroño | End | N 19°28.617' W 98°35.278' | N 19°28.593' W 98°35.352' | N 19°28.591' W 98°35.382' |
| 6 | 11. Ejido San Gabriel | Start | N 19°15.610' W 98°37.260' | N 19°15.586' W 98°37.161' | N 19°15.536' W 98°37.267' |
| 6 | 11. Ejido San Gabriel | Middle | N 19°15.599' W 98°37.257' | N 19°15.583' W 98°37.176' | N 19°15.549' W 98°37.265' |
| 6 | 11. Ejido San Gabriel | End | N 19°15.587' W 98°37.254' | N 19°15.583' W 98°37.176' | N 19°15.558' W 98°37.261' |
| 6 | 12. San Rafael Ixtapalucan 2 | Start | N 19°16.571' W 98°36.644' | N 19°16.542' W 98°36.622' | N 19°16.599' W 98°36.601' |
| 6 | 12. San Rafael Ixtapalucan 2 | Middle | N 19°16.555' W 98°36.651' | N 19°16.554' W 98°36.617' | N 19°16.593' W 98°36.587' |
| 6 | 12. San Rafael Ixtapalucan 2 | End | N 19°16.552' W 98°36.650' | 19°16.561' W 98°36.615' | N 19°16.589' W 98°36.569' |

**Table S2.** Significant environmental variables (*p* < 0.05) resulted from the redundancy analysis (RDA) on the Hellinger-transformed dataset using Horn distance.

| Environmental variable | Geneious+  UNITE | Single MiCoP | Paired MiCoP | Kraken2 |
| --- | --- | --- | --- | --- |
| Ca content | 0.005 | - | - | - |
| WHC ^a^ | 0.005 | 0.030 | - | - |
| OM content ^b^ | 0.005 | - | - | 0.005 |
| pH | 0.005 | 0.005 | - | 0.010 |
| Mg content | **-** | - | - | - |
| N content | - | 0.005 | - | - |
| Fe content | - | - | - | 0.025 |
| ^a^ WHC: water holding capacity; OM: ^b^ Organic material. | | | | |

**Table S3.** Significant environmental variables (*p* < 0.05) determined with a redundancy analysis (RDA) with the Hellinger-transformed using Bray-Curtis distance.

| Environmental variable | Geneious+  UNITE | Single MiCoP | Paired MiCoP | Kraken2 |
| --- | --- | --- | --- | --- |
| Ca content | 0.005 | 0.005 | - | 0.005 |
| WHC ^a^ | 0.005 | 0.015 | - | - |
| OM content ^b^ | 0.005 | - | - | - |
| pH | 0.005 | 0.005 | - | 0.005 |
| Mg content | - | 0.005 | 0.035 | - |
| N content | - | - | 0.025 | - |
| Cu content | - | - | - | 0.005 |
| P content | - | - | - | 0.020 |
| Fe content | - | - | - | 0.030 |
| ^a^ WHC: water holding capacity; OM: ^b^ Organic material | | | | |

**Table S4.** Significant vegetation traits (*p* < 0.05) determined with a redundancy analysis (RDA) with the Hellinger-transformed using Horn distance.

| Vegetation variable | Geneious+  UNITE | Single MiCoP | Paired MiCoP | Kraken 2 |
| --- | --- | --- | --- | --- |
| Proportion of *Arbutus* | 0.005 | - | - | - |
| Proportion of *Abies* | - | 0.005 | - | - |
| Proportion of *Pinus* | - | 0.005 | - | - |
| Proportion of *Alnus* | 0.015 | 0.020 | 0.040 | - |
| Total height | - | - | - | - |
| Coverage of *Pinus* | - | 0.005 | - | - |
| Total coverage | - | - | 0.040 | - |
| Proportion of *Salix* | - | - | - | 0.015 |
|  |  |  |  |  |

**Table S5.** Significant vegetation traits (*p* < 0.05) determined with a redundancy analysis (RDA) with the Hellinger-transformed using Bray-Curtis distance.

| Vegetation variable | Geneious+UNITE | Single MiCoP | Paired MiCoP | Kraken 2 |
| --- | --- | --- | --- | --- |
| Proportion of *Arbutus* | 0.005 | - | - | - |
| Proportion of *Abies* | - | 0.005 | 0.005 | - |
| Proportion of *Pinus* | 0.005 | 0.005 | - | - |
| Proportion of *Alnus* | 0.030 | 0.005 | 0.015 | - |
| Total height | - | - | - | - |
| Proportion of broadleaf | - | - | 0.020 | - |
| Proportion of *Salix* | - | - | - | 0.025 |
| Proportion of *Juniperus* | - | - | - | 0.005 |
| Total coverage | - | - | - | 0.010 |
|  |  |  |  |  |

**Table S6.** Partial Mantel test of correlations between community distances matrices (Horn dissimilarity) and environmental Euclidean distances controlled by spatial coordinates.

|  | Geneious+UNITE | | Single  MiCoP | | Paired  MiCoP | | Kraken 2 | |
| --- | --- | --- | --- | --- | --- | --- | --- | --- |
|  | **r** | **p-value** | **r** | **p-value** | **r** | **p-value** | **r** | **p-value** |
| Environmental | 0.260 | **0.006** | 0.182 | **0.036** | 0.112 | 0.09 | 0.192 | **0.015** |
| Vegetation | -0.069 | 0.751 | 0.140 | 0.126 | -0.086 | 0.796 | 0.117 | 0.112 |
|  |  |  |  |  |  |  |  |  |

**Table S7.** Partial Mantel test of correlations between community distances matrices (Bray-Curtis dissimilarity) and vegetation Euclidean distances controlled by spatial coordinates.

|  | | Geneious+  UNITE | | | Single  MiCoP | | | Paired  MiCoP | | | Kraken 2 | |
| --- | --- | --- | --- | --- | --- | --- | --- | --- | --- | --- | --- | --- |
|  | **r** | | **p-value** | **r** | | **p-value** | **r** | | **p-value** | **r** | | **p-value** |
| Environmental | 0.280 | | **0.001** | 0.203 | | **0.024** | 0.164 | | 0.053 | 0.196 | | **0.010** |
| Vegetation | -0.038 | | 0.621 | 0.056 | | 0.281 | -0.098 | | 0.793 | 0.178 | | **0.036** |

**Table S8.** Summary of the frequencies obtained with Kraken2 fungi database

| Bioinformatic workflow | Total  frequencies | Number of  Features | Minimum  frequency  by sample | Maximum  frequency  by sample | Mean  frequency  by sample | Median  frequency  by sample | Mean  Good’s coverage (%) |
| --- | --- | --- | --- | --- | --- | --- | --- |
| Kraken2-  fungi | 2,132,427 | 72 | 420,064 | 752,161 | 592,340 | 594,977 | 100 |

**Fig. S1** Boxplots with alpha diversities (Hill numbers at *q* = 0, 1 and 2) of fungal taxonomic species or operational taxonomic units at 97% of similarity (features) obtained with different bioinformatic workflows (see materials and methods). (a-c) GENEIOUS+UNITE, (d-f) SINGLE MICOP, (g-i) PAIRED MICOP, and (j-l) KRAKEN2 across the twelve sampling sites (1-12). The Kruskal-Wallis test was used to compare the different sites (significant at *p* < 0.05). Colored numbers refer to the different sampling sites and their location is given in Figure 1.
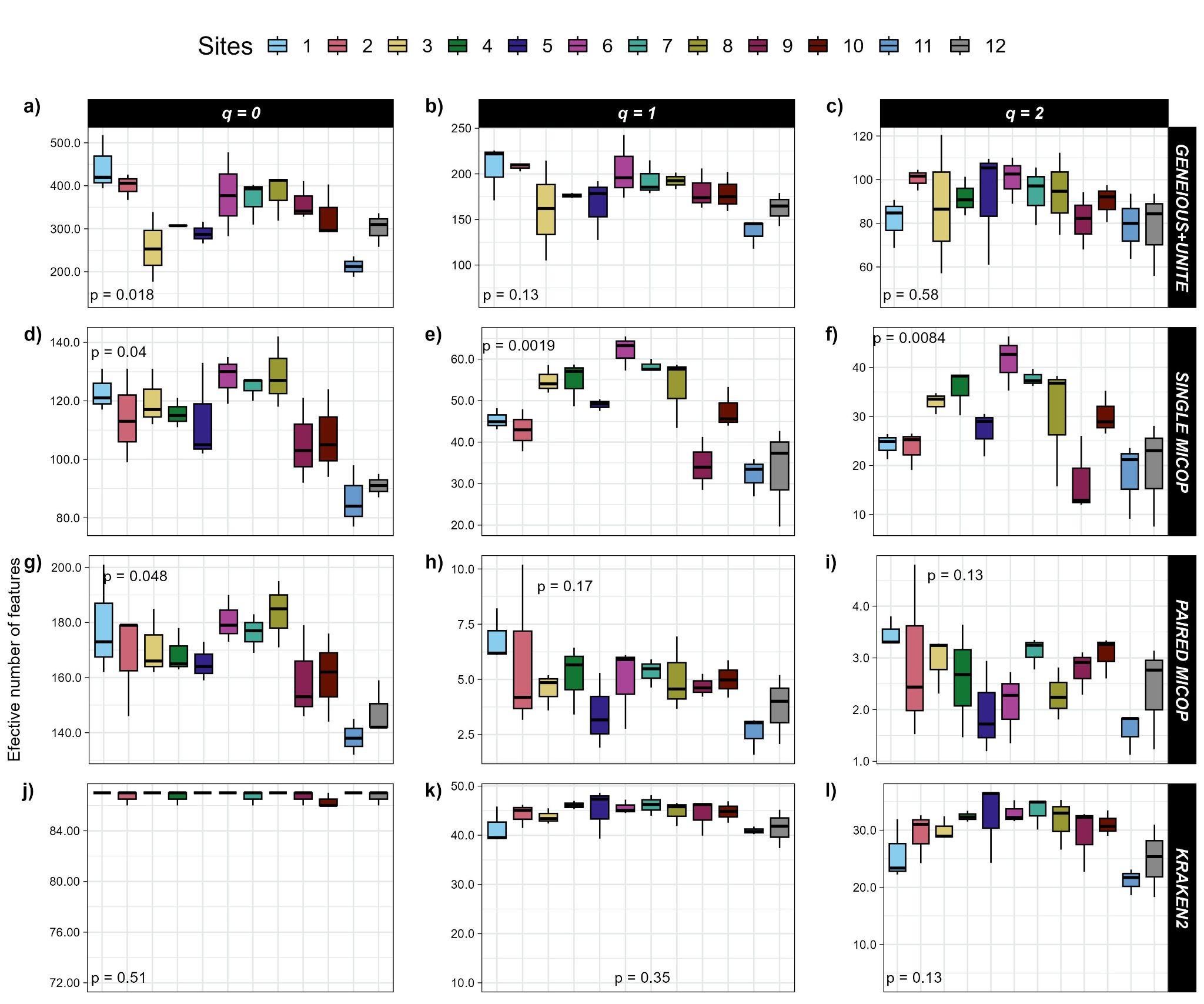


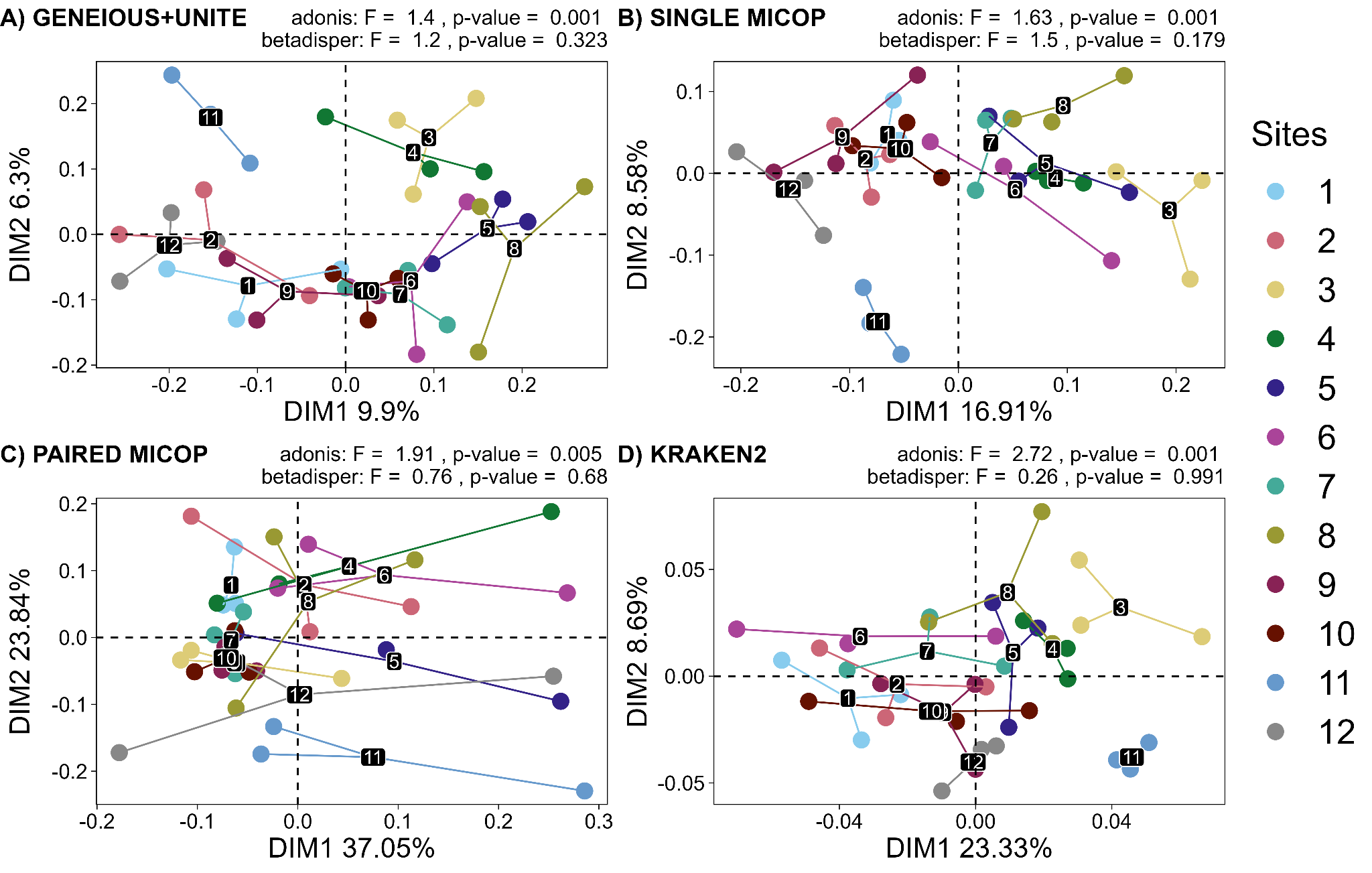


**Fig. S2** Unconstrained principal coordinate analysis (PCoA) with Bray-Curtis dissimilarities of the taxonomic structure of fungal communities (operational taxonomic units at 97% similarity or species) using different bioinformatic workflows: A) GENEIOUS+UNITE, B) SINGLE MICOP, C) PAIRED MICOP, and D) KRAKEN2 (see Figure 2). Variations in variance and homogeneity among sampling sites were determined with a permutational analysis of variance (adonis) and a permutational multivariate homogeneity of group dispersions analysis (betadisper). Numbers refer to the different sites and their location is given in Figure 1.

**
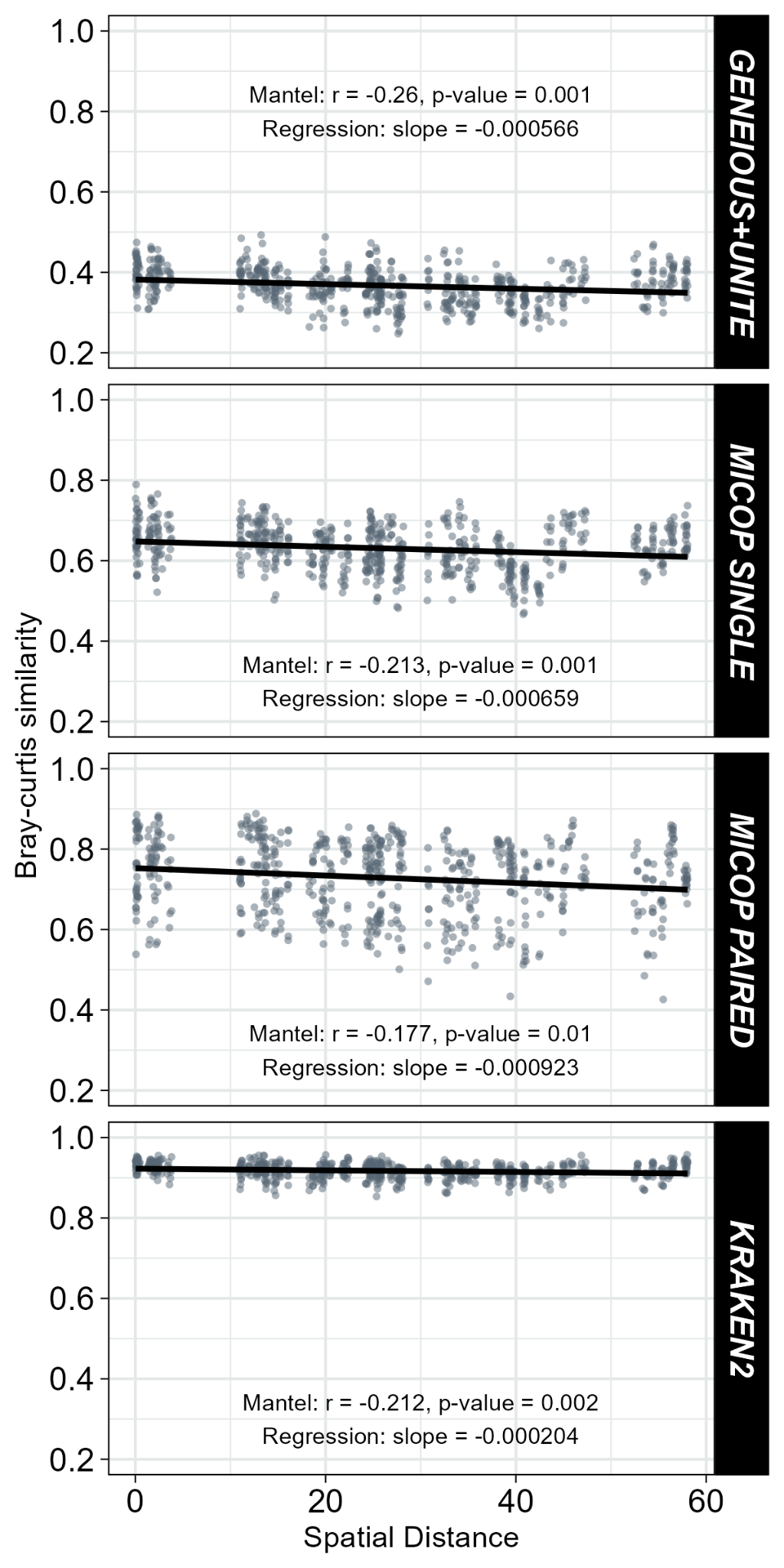
**

**Fig. S3** Relationship between Bray-Curtis similarity of fungal communities obtained by different bioinformatic workflows (GENEIOUS+UNITE, SINGLE MICOP, PAIRED MICOP, and KRAKEN2; see Figure 2) and geographical distance. Pearson’s correlation tests were done to determine the association between community similarity and geographic matrices.


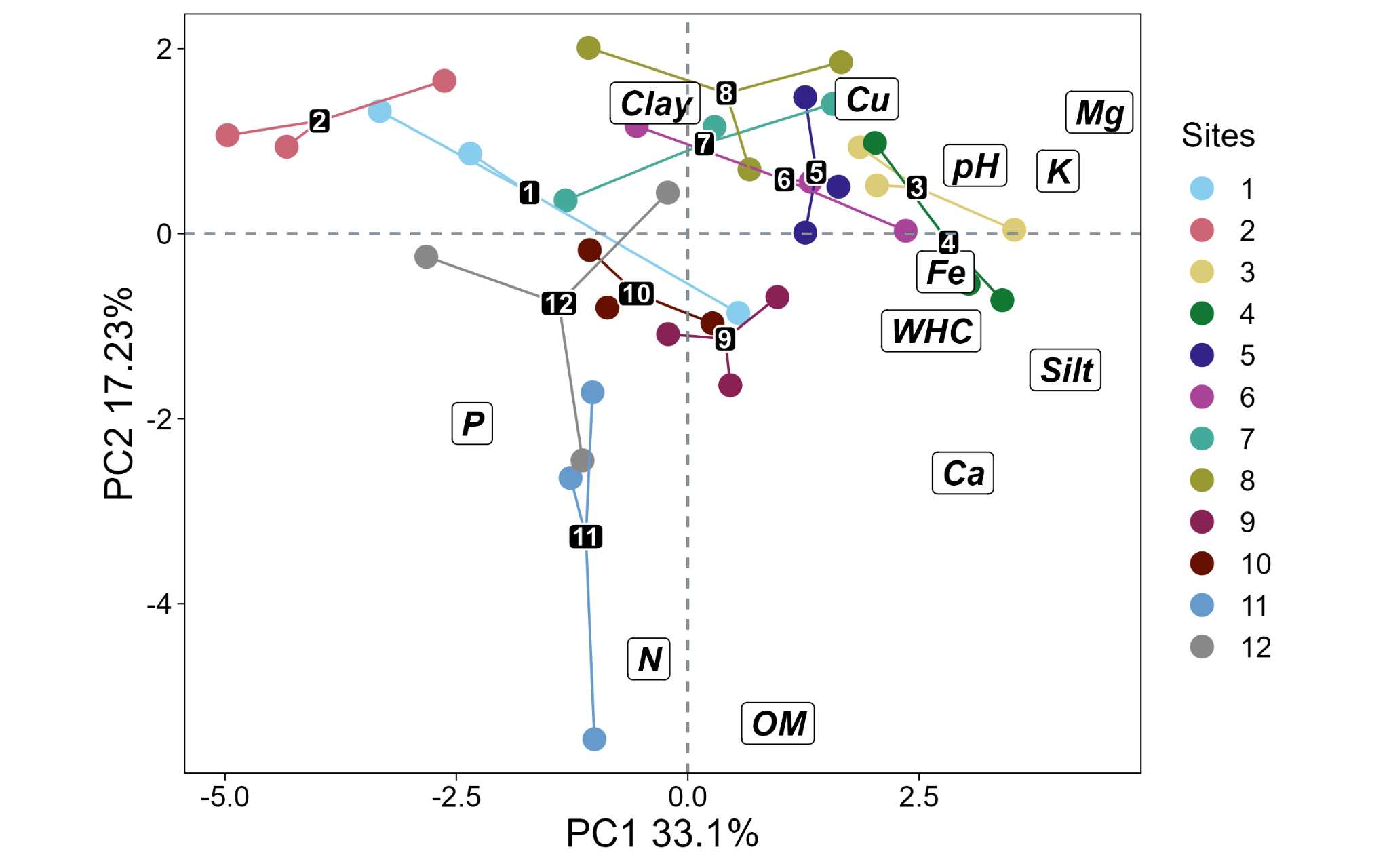


**Fig. S4** Principal component analysis (PCA) of environmental characteristics, i.e, physicochemical soil properties and nutrient content by sampling sites. Numbers refer to the different sampling sites and their location is given in Figure 1. WHC: water holding capacity, SOM: soil organic matter.


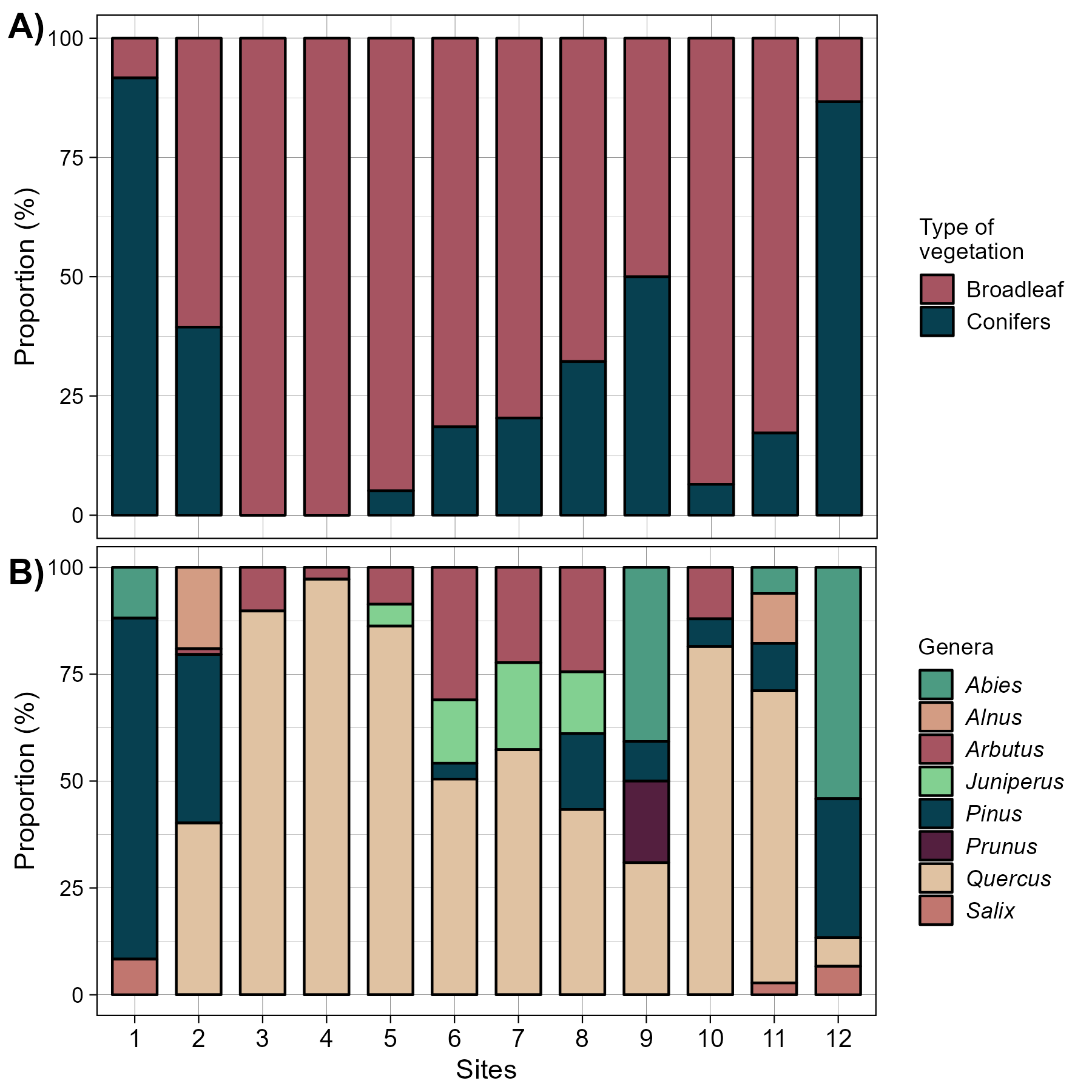


**Fig. S5** Barplots with the proportions of A) vegetation type, i.e, conifer and broadleaf; and B) by genera across sites of sampling. Numbers in the x-axis refer to the different sampling sites and their location is given in Figure 1.


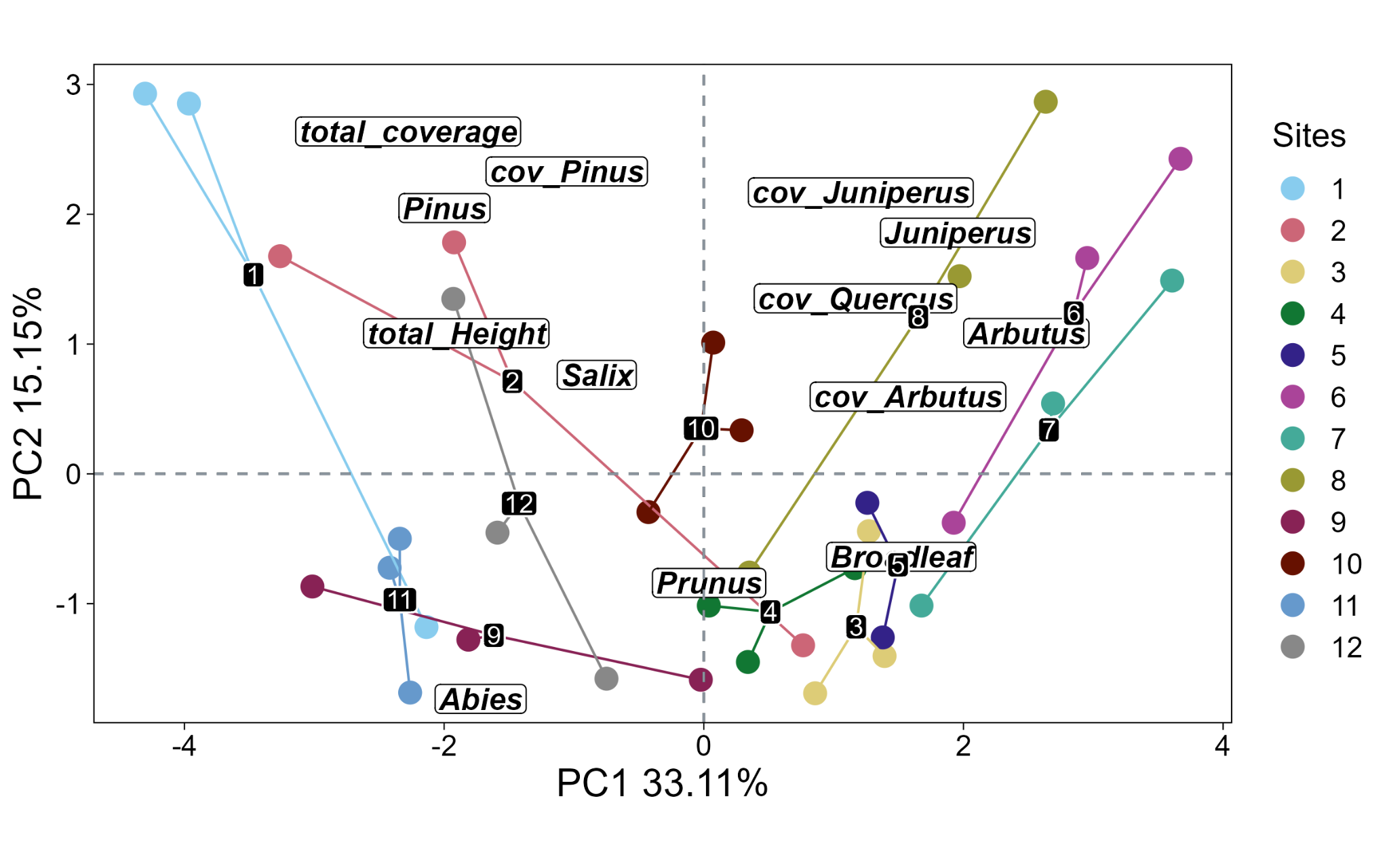


**Fig. S6** Principal component analysis (PCA) of vegetation and its traits by sampling sites. Colored numbers refer to the different sampling sites and their location is given in Figure 1.

**
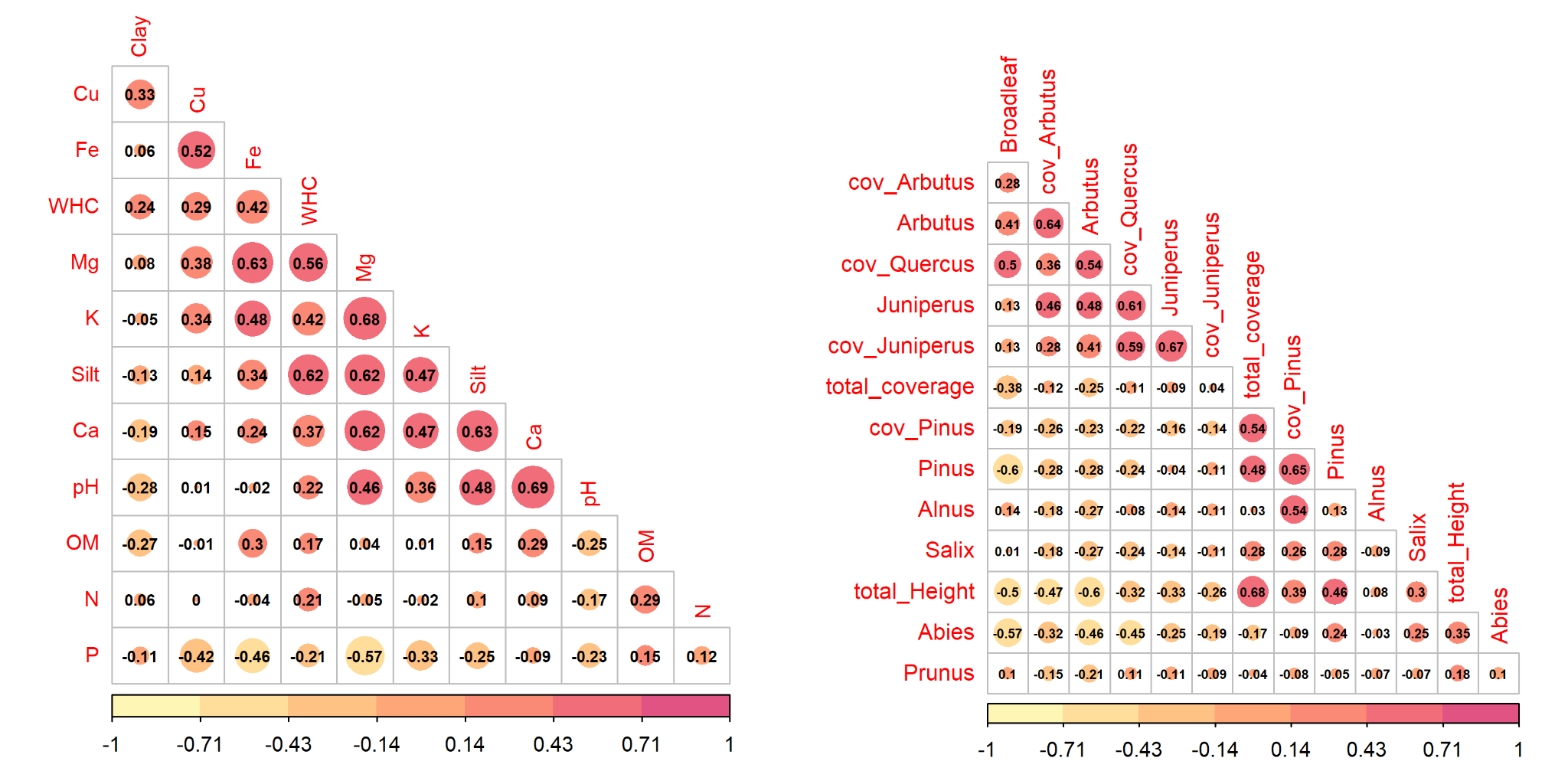
**

**Fig. S7.** Pearson correlation coefficient between soil characteristics and vegetation characteristics. Variables that were highly correlated (r > |0.7|) are not given in the figure. SOM: organic material; WHC: water holding capacity; cov: coverage.


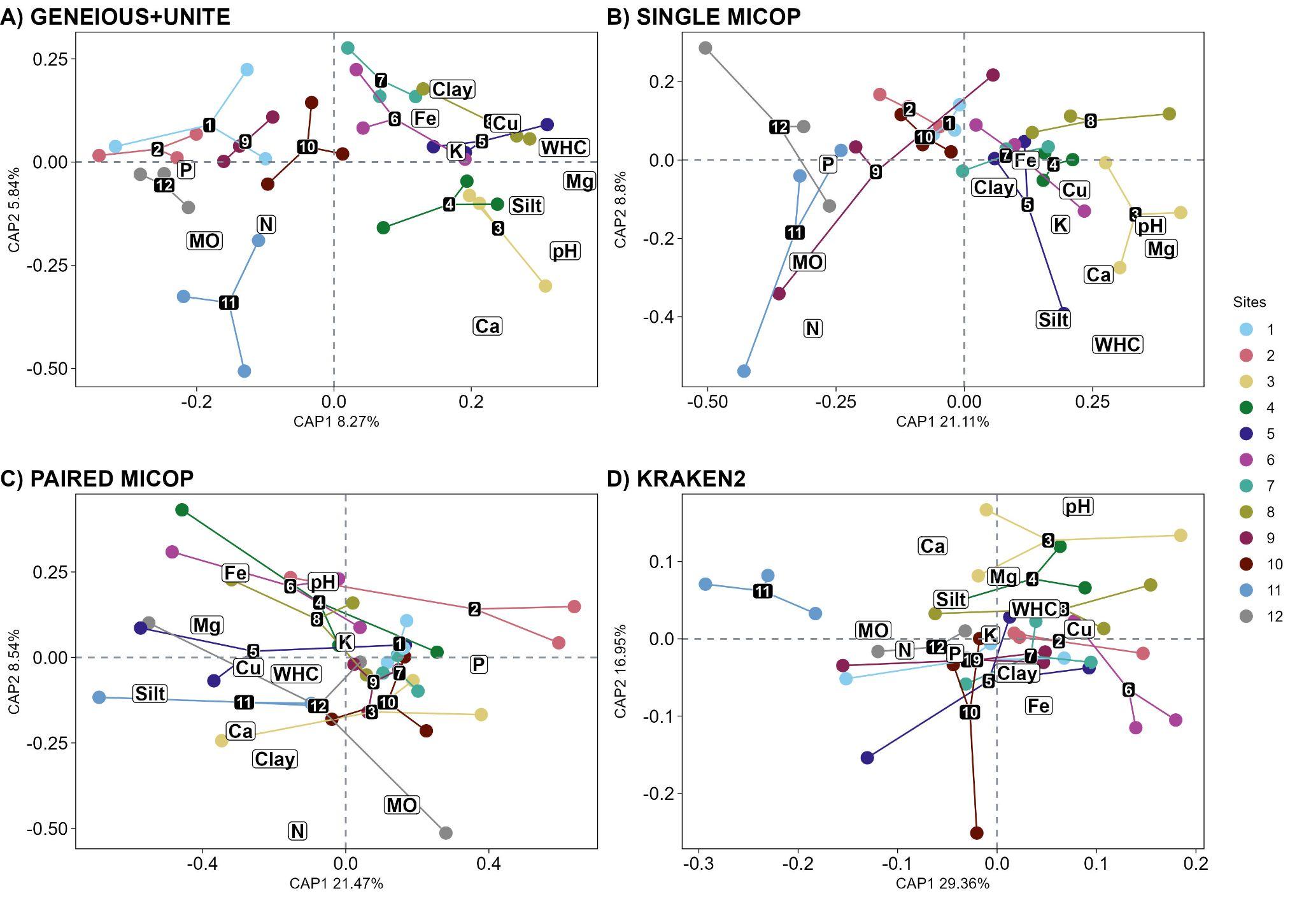


**Fig. S8.** Canonical analysis of principal coordinates (CAPS) with the Hellinger-transformed sequence data using Horn distance (accounting for abundance) across different methods using environmental variables. Different workflows for taxonomic profiling were used: A) GENEIOUS+UNITE, B) SINGLE MICOP, C) PAIRED MICOP, and D) KRAKEN2 (see Figure 2). Numbers refer to the different sampling sites and their location is given in Figure 1. WHC: water holding capacity, SOM: soil organic matter.


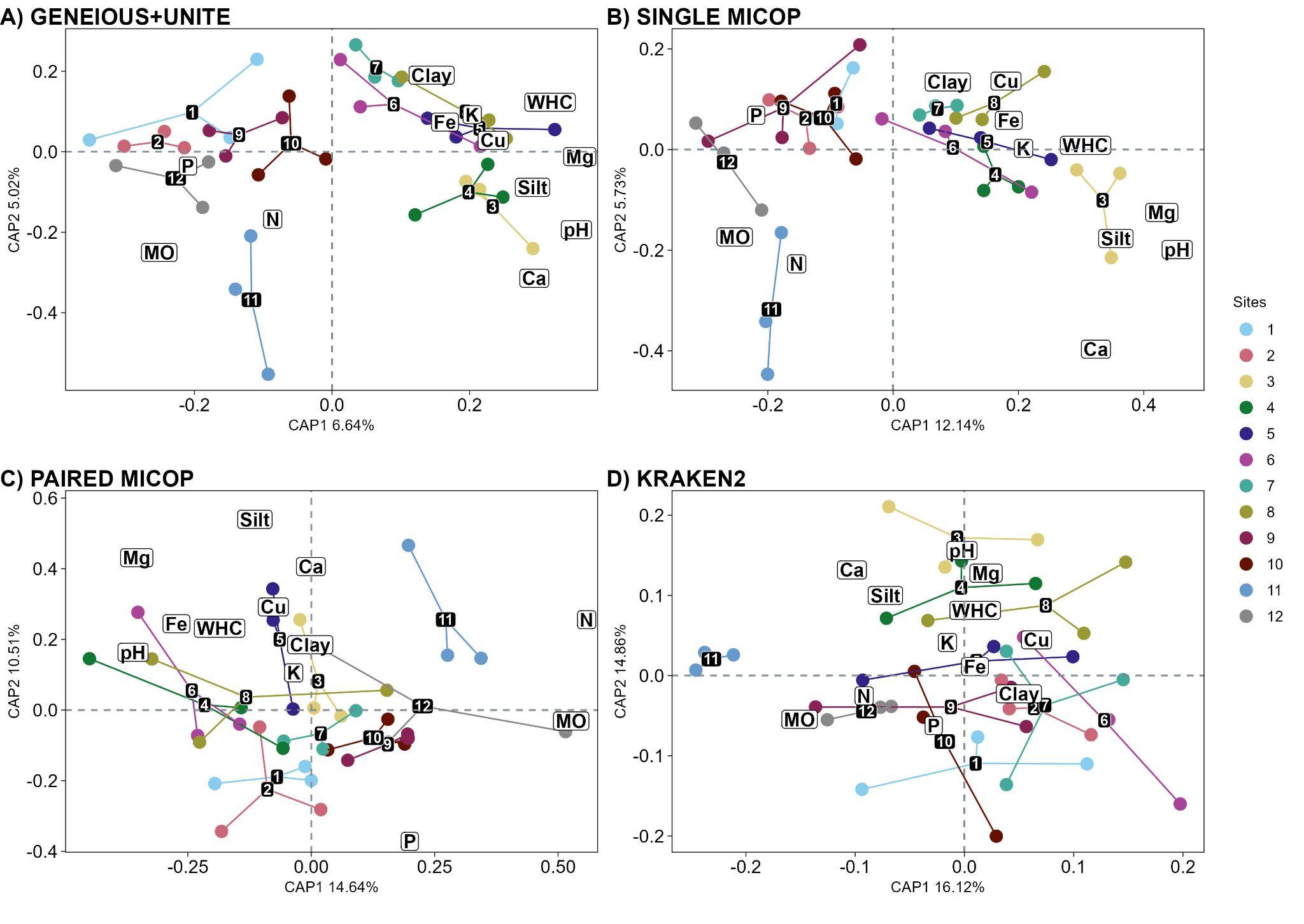


**Fig. S9.** Canonical analysis of principal coordinates (CAPS) with the Hellinger-transformed sequence data using Bray-Curtis distance (accounting for abundance) across different methods using environmental variables. Different workflows for taxonomic profiling were used: A) GENEIOUS+UNITE, B) SINGLE MICOP, C) PAIRED MICOP, and D) KRAKEN2 (see Figure 2). Numbers refer to the different sampling sites and their location is given in Figure 1. WHC: water holding capacity, SOM: soil organic matter.


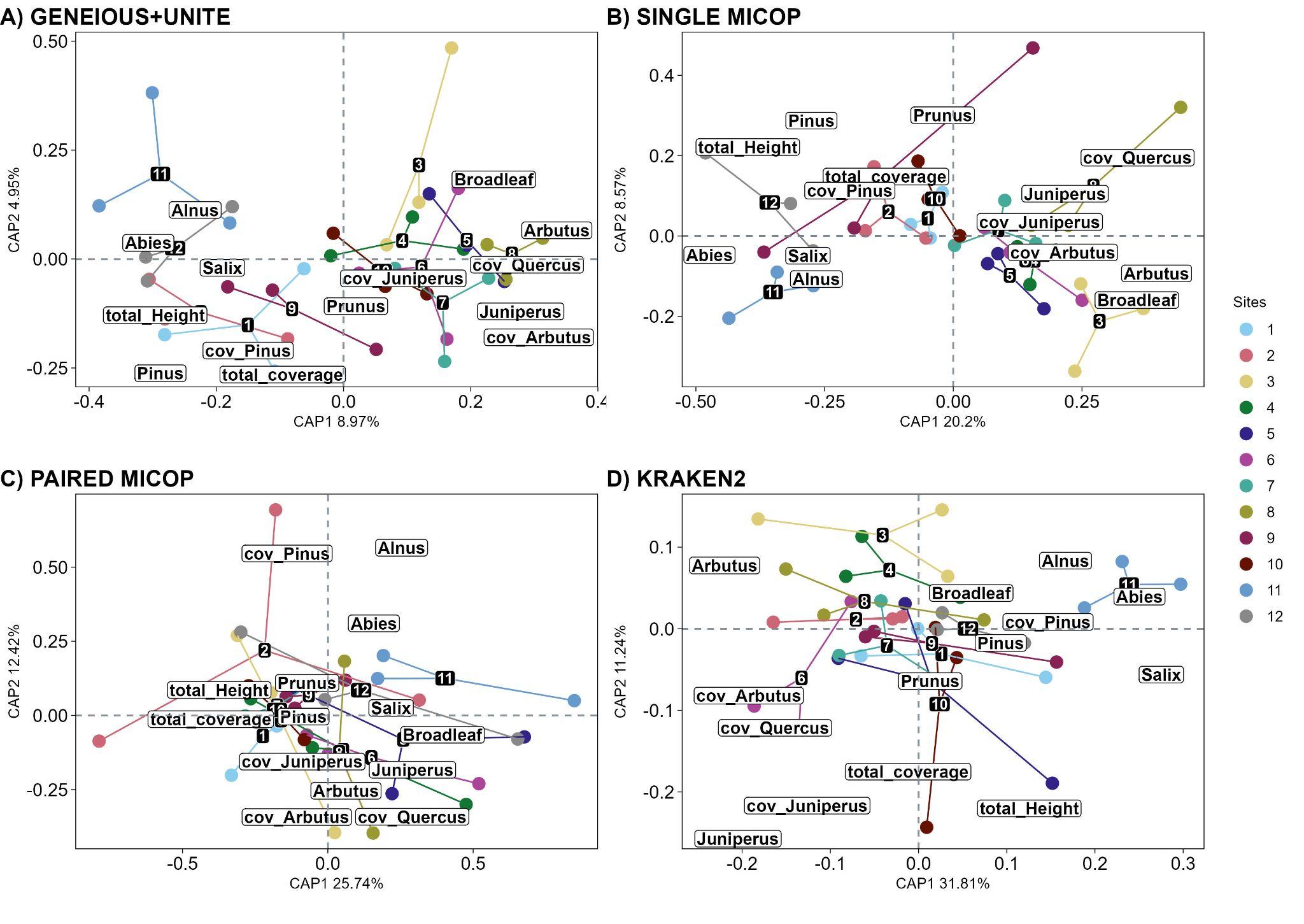


**Fig. S10.** Canonical analysis of principal coordinates (CAPS) with the Hellinger-transformed sequence data using Horn distance (counting for abundance) across different methods using vegetation variables. Different workflows for taxonomic profiling were used: A) GENEIOUS+UNITE, B) SINGLE MICOP, C) PAIRED MICOP, and D) KRAKEN2 (see Figure 2). Numbers refer to the different sampling sites and their location is given in Figure 1. Cov: coverage.


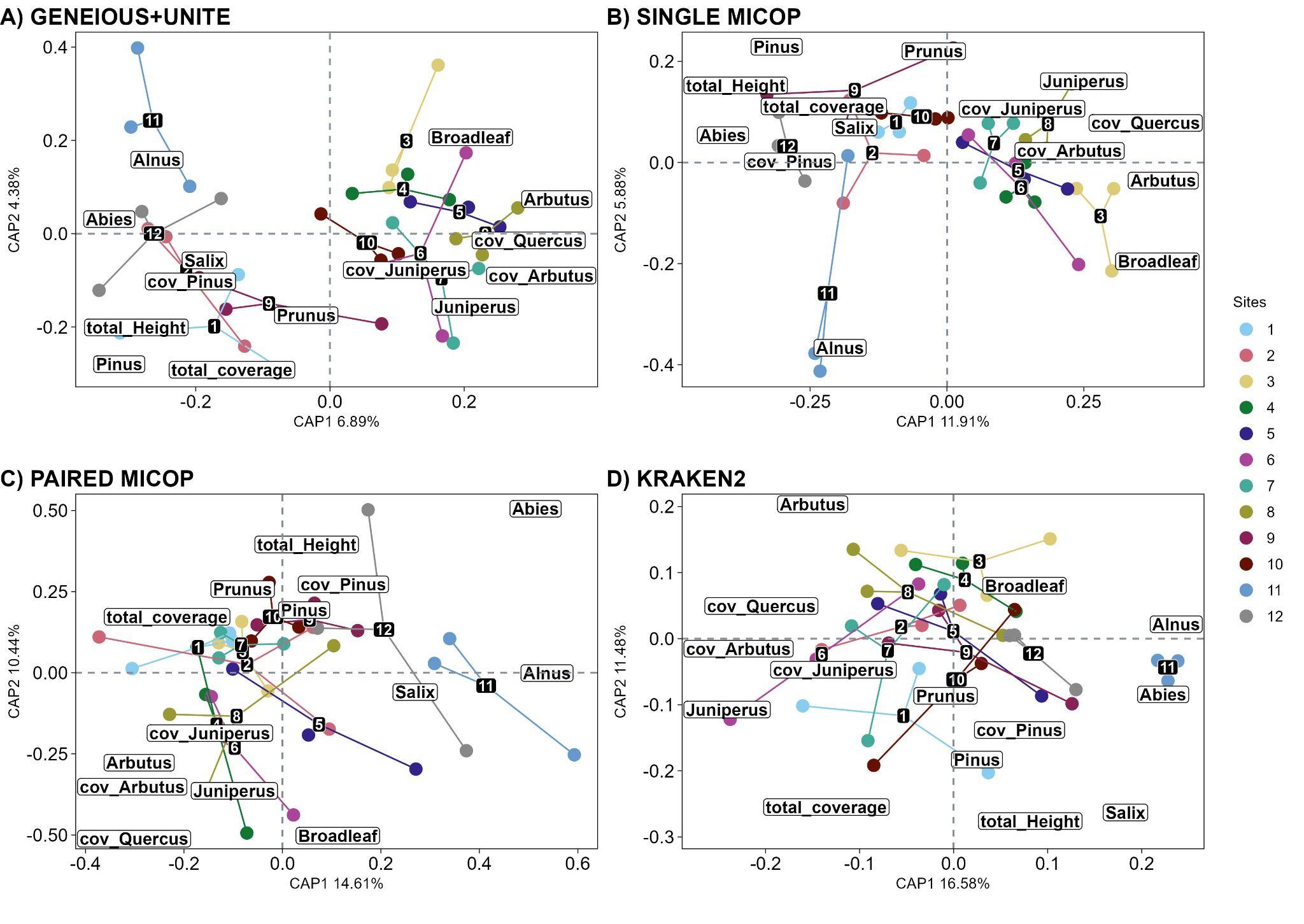


**Fig. S11.** Canonical analysis of principal coordinates (CAPS) with the Hellinger-transformed sequence data using Bray-Curtis distance (counting for abundance) across different methods using vegetation variables. Different workflows for taxonomic profiling were used: A) GENEIOUS+UNITE, B) SINGLE MICOP, C) PAIRED MICOP, and D) KRAKEN2 (see Figure 2). Numbers refer to the different sampling sites and their location is given in Figure 1. Cov: coverage.
